# The Value of Helicobacter Eradication in Long-term Aspirin Users

**DOI:** 10.1093/jnci/djx289

**Published:** 2018-01-19

**Authors:** Jack Cuzick

**Affiliations:** Wolfson Institute of Preventive Medicine, Queen Mary University of London, London, UK

In this issue, Cheung et al. (1) report a surprisingly large preventive effect of aspirin on gastric cancer in individuals who have been successfully treated for *Helicobacter pylori*. Most of the more than 50 randomized trials and 100 epidemiologic studies examining the impact of aspirin use on gastric cancer reported a reduction of 30% to 35% in incidence and mortality among long-term users, with little impact in the first three to five years of use (2–5). However, most of these studies have not examined the effect of aspirin according to *H. pylori* status, and the ones that have (6–8) do not clearly separate those where the infection was successfully treated from those where it was not. The current report only considers those who were *H. pylori* positive but in whom the infection was successfully treated, and it suggests that aspirin use has led to a 70% (95% confidence interval [CI] = 39% to 85%) reduction in gastric cancer in this group.

Although the incidence and mortality from gastric cancer have fallen dramatically in the last century, worldwide it is still the fifth commonest cancer with the third highest mortality rate, accounting for 952 000 cases and 723 000 deaths (9). There is a large geographic variation in gastric cancer, mostly due to variations in noncardia cancers, with much less variation in cardia (top section of the stomach adjoining the esophagus) gastric cancer (10). Cardia cancers account for only 4% of gastric cancer in Japanese males compared with 39% of white males in the United States (10). Plummer et al. (11) estimate that almost 90% of noncardia cancers are attributable to *H. pylori* infection as a result of it causing atrophic gastritis (12). It appears to have no impact on cardia gastric cancers, and the attributable risk for all gastric cancers has been estimated at between 65% and 80%, depending on geographic location (13). Two recent overviews of studies of eradication of *H. pylori* using different studies and different selection criteria have reported a 44% reduction (14) and a 33% reduction (15) in gastric cancer, respectively. However, there is some suggestion that eradication could increase reflux and cardia cancers (16). Both gastric cancer and *H. pylori* infection are more common in Eastern Asia and South America than in the rest of the world, with sixfold higher gastric cancer rates in Eastern Asia than in the United States (9). *H. pylori* infection rates are estimated to be about 35% in the United States vs 55% in Asia (17), but curiously they are only slightly higher in men than women (odds ratio [OR] = 1.12, 95% CI = 1.09 to 1.15) (18), despite the fact that gastric cancer is about twice as common in men compared with women. Other gastric cancer risk factors include family history, tobacco smoking, high intake of salty food, and low intake of fruits and vegetables (13).

Recent data have suggested that daily use of low-dose aspirin for 10 years between the ages of 50 years and 70 years could reduce the total cancer burden by 7% to 10% in the next 15 years and cancer mortality by between 9% and 13% in the next 20 years (5). The major impact is on three gastrointestinal (GI) cancers—colorectal, gastric, and esophageal, where the reductions are approximately 30% for each. The main side effect of aspirin use is gastrointestinal bleeding. The rates increase with age and are higher in men. Aspirin use increases this by about 60%, leading to an approximately 0.7% absolute increase in major bleeds over a 10-year period for men age 60 to 70 years (19,20).

*H. pylori* infection is a major risk factor, both for GI bleeding and for gastric cancer. Several models have suggested that population screening would be cost-effective, and in their recent report the International Agency for Research on Cancer (IARC) Working Group “recommends that countries explore the possibility of introducing population-based *H. pylori* screening and treatment programmes, but cautions that decisions as to whether and how to implement *H. pylori* testing and treatment must hinge on local considerations of disease burden, other health priorities, and cost–effectiveness analyses” (21). In the United States and United Kingdom, *H. pylori* testing is currently only recommended for those with symptoms of gastric or duodenal ulcers.

*H pylori* testing would seem particularly important for those taking aspirin on a regular basis, and should further improve the already substantial benefit-harm ratio associated with prophylactic aspirin use. In the United Kingdom, the HEAT trial is currently evaluating the impact of *H. pylori* testing and eradication on GI bleeding in current long-term low-dose aspirin users (22).

The study by Cheung et al. suggests that the effect of aspirin in preventing gastric cancer could be even larger if *H. pylori* was eradicated before its use. However, there are several caveats in interpreting these results. First, the number of cases in aspirin users in this study is small. Overall, 25 cases were reported in the 9045 aspirin users, but only 12 gastric cancers were reported in those with at least weekly use (n = 6466) and no benefit was seen in less frequent users. Additionally, no benefit was seen for users of duration of less than two years, and only six cancers were reported in the 5354 users for two years or longer, which included an unreported number of less than weekly users. The effect size for regular long-term users is not reported, but confidence intervals will be wide.

The main results are corrected for a propensity index to allow for the substantially different age and sex distribution in aspirin users vs nonusers, as well as other poorly recorded risk factors for gastric cancer such as smoking. No details were provided on the weights of specific variables in the propensity index or the specific method for incorporating them into the analysis, as several methods with differing potential biases are possible (23). As no aspirin effect was seen in the uncorrected analyses, it would have been useful to also report the hazard ratio adjusted only for age and sex, which would probably adjust for most of the confounding in a more transparent and straightforward manner.

Another concern is that *H. pylori* testing was not done routinely as a screening procedure, but probably mostly in symptomatic individuals, and whether the effect of aspirin is similar in those who are untreated asymptomatic positives or who have never been infected is not addressed here, as they will be the majority in Western populations.

In summary, the results from Cheung et al. indicate that there is no loss of aspirin’s risk reduction effect for gastric cancer when *H. pylori* is tested for and eradicated. It would be reassuring to also see data for those who tested negative, and for other GI cancers for which aspirin has been shown to be preventive, although it seems likely that there would not be an effect of *H. pylori* on them. As *H. pylori* infection is still quite prevalent among middle-aged individuals throughout the world and GI bleeding is the major side effect of regular aspirin use, these results suggest that *Helicobacter pylori* testing and eradication should be considered before commencement of long-term prophylactic aspirin use for cancer or cardiovascular disease prevention.

## Note

The author is involved in a pilot trial of aspirin use in early prostate cancer managed by active surveillance. Bayer provided free aspirin for use in this trial.
